# Correction: Computational analysis of the deleterious non-synonymous single nucleotide polymorphisms (nsSNPs) in TYR gene impacting human tyrosinase protein and the protein stability

**DOI:** 10.1371/journal.pone.0321098

**Published:** 2025-03-25

**Authors:** Wei Fan, Heng Li Ji, Mohibullah Kakar, Shabbir Ahmed, Hussah M. Alobaid, Yasmeen Shakir

There are errors in the Funding statement. The correct Funding statement is as follows: This work is funded by the Research Supporting Project Number (RSP2025R174), King Saud University, Riyadh, Saudi Arabia.

## References

[1] Fan W, Ji HL, Kakar M, Ahmed S, Alobaid HM, Shakir Y. Computational analysis of the deleterious non-synonymous single nucleotide polymorphisms (nsSNPs) in TYR gene impacting human tyrosinase protein and the protein stability. PLoS One. 2024;19(11): e0308927. doi: 10.1371/journal.pone.0308927 39541331 PMC11563463

