# Supplementary material for: Computational analysis of the deleterious non-synonymous single nucleotide polymorphisms (nsSNPs) in TYR gene impacting human tyrosinase protein and the protein stability
Source: PLoS One. 2024 Nov 14;19(11):e0308927. doi: 10.1371/journal.pone.0308927 (PMC11563463; doi:10.1371/journal.pone.0308927)
Supplement: S1 Table — (DOCX) [file pone.0308927.s001.docx]

**S1 Table:** Reported the most deleterious variants decreasing the protein stability while present in the highly conserved region of the Tyrosinase protein.
