# Supplementary material for: Computational analysis of the deleterious non-synonymous single nucleotide polymorphisms (nsSNPs) in TYR gene impacting human tyrosinase protein and the protein stability
Source: PLoS One. 2024 Nov 14;19(11):e0308927. doi: 10.1371/journal.pone.0308927 (PMC11563463; doi:10.1371/journal.pone.0308927)
Supplement: Attachment — Submitted filename: Response latter.docx [file pone.0308927.s002.docx]

**Response latter**

********

August 24^th^ 2023

**Dear Editor in Chief**

**PLOS ONE**

**After greeting**

I'd like to extend my thanks and appreciation for your kind efforts. This is regarding my manuscript Ref. **Submission ID PONE-D-23-24946**, entitled "**Computational analysis of damaging non-synonymous single nucleotide polymorphisms (nsSNPs) on human tyrosinase protein**". I confirm that all corrections, suggestions and queries made by the editor were all given full consideration. All corrections were made in the manuscript file and marked as track change. Our response against each recommendations is mentioned below.

**Additional Editor Comments:**
-The corresponding author doesn't match in the manuscript.docx copy with a manuscript draft form, it caused confusion.

Response: Corrected
-The whole manuscript needs native-language editing.

Response: We have revised the language of manuscript with help the help of expert colleague.
-Some texts are not based on the reference. ex. Line no. 89–90:

Response: Revised
-Line no. 29–93: The authors stated ''to date'' although citing this number and information from a reference published in 2001.

Response: Revised
-Line no. 38: keywords was not covering all input keys.

Response: Revised

The authors of this manuscript are grateful to the subject expert and editorial members of the journal, for valuable suggestions to improve the quality of our manuscript. We hope this version is suitable for acceptance and publication.

_________________________

**Muhammad Asif Arain**

Corresponding author

asifarain77@yahoo.com
