# Supplementary material for: Computational analysis of the deleterious non-synonymous single nucleotide polymorphisms (nsSNPs) in TYR gene impacting human tyrosinase protein and the protein stability
Source: PLoS One. 2024 Nov 14;19(11):e0308927. doi: 10.1371/journal.pone.0308927 (PMC11563463; doi:10.1371/journal.pone.0308927)
Supplement: Attachment — Submitted filename: Plos One Review Report.docx [file pone.0308927.s003.docx]

***Review Report***

***Computational analysis of damaging non-synonymous single nucleotide polymorphisms (nsSNPs) on human tyrosinase protein***

The above manuscript is interesting in describing the structural and functional consequences of disease-associated non-synonymous single nucleotide polymorphisms (nsSNPs) in the TYR gene, which encodes tyrosinase. The study highlights the nsSNPs, notably K142M, I151N, M179R, S184L, L189P, and C321R, as particularly deleterious variants, which in general impact the structural integrity and functional behavior of human tyrosinase. The target is of importance; however, as a computational biologist, I am not confident about the outcomes of this study due to a limited and primary-level methodology. Following are some suggestions that need to be addressed before the final decision on the manuscript:

1. The title is very classical; preferably, it should be reworded. Specifically, the main components of the title should be rephrased with some smart, appealing words.
2. Abstract: The abstract is very plain and incomplete, lacking key content areas, and the research purpose was not properly addressed. The relevance or importance of the research work and the main outcomes were not discussed adequately. See lines 31–35; these lines seem to be an incorrect piece of information. It should be explained in detail regarding the methodological scope. What is the purpose of mentioning lines 37–38? Please explain. Please justify how this study advances the readers’ understanding of the molecular repercussions of the target’s gene and its variants.
3. Keywords: The keywords were not appropriate. Please recheck all selected words.
4. Introduction: This section looks like a mismatch of information. The introduction needs a thorough revision, especially in terms of describing the background of the gene or protein target selected, its clinical role, and its recent epidemiological impact on society. I absolutely missed thorough literature that represents a sound background of information from the present work. I would suggest here that authors should make this section a more comprehensive and updated flow of information that makes readers understand the importance of selected genes and why authors used them for this in silico genomic study. The study rational was missing from this section. Why are authors emphasizing that the main aim of this study is to see the significant role of non-synonymous genetic variations and their impact on the tyrosinase protein`s structure and function.? Specifically, the last paragraph was quite confusing and lacked clarity. The abstract isn’t organized and looks more like a mishmash of information.
5. Methodology: The very first sentence starts with “to date." I think it is better to mention the search timeline period. This section was absolutely incomplete. It is quite better if authors segregate the prediction methodologies separately in sub-headings. More importantly, this sub-division will help authors mention gene- or protein-based, sequence- or structure-based assessment methodologies. I found some repetition of details in this section. Although this is an optional suggestion, authors should add a flow chart with a better and more appealing way of presenting it. Please check the following manuscripts for a better reference.
6. DOI: 10.1021/acsomega.2c04871
7. DOI: 10.1021/acsptsci.2c00212
8. DOI: 10.1021/acs.jcim.0c00488
9. Results: I don’t accept the results in their present form. The results should be divided and subdivided according to the work scope. These aren’t acceptable in the present form. The graphical representations in the manuscript are of extremely low quality, and the authors are advised to use some smart tools to render high-quality, eye-catching plots of their data.
10. Discussion: It would be better to revise this section. Don’t provide too many details about the tools and methods used in the manuscript; focus more on whether your results are consistent and contribute to your overall findings in relation to previous studies and, finally, your future prospects.
11. Citations are missing in some cases.
12. Add a proper conclusion section to help readers retain the purpose of the article.

**Minor comments:**

1. Cover letter: Please check the name and details of the corresponding author mentioned in the reply to the review letter. It should be the same as listed in the authors’ name list of this paper.
2. Kindly add some missing references.
3. Please refer to the above-recommended manuscripts for guidance on writing, figures, and work methodology.

Good luck!
