# Supplementary material for: Computational analysis of the deleterious non-synonymous single nucleotide polymorphisms (nsSNPs) in TYR gene impacting human tyrosinase protein and the protein stability
Source: PLoS One. 2024 Nov 14;19(11):e0308927. doi: 10.1371/journal.pone.0308927 (PMC11563463; doi:10.1371/journal.pone.0308927)
Supplement: Attachment — Submitted filename: Response latter.docx [file pone.0308927.s004.docx]

**Response latter**

********

February 13^th^ 2024

**Dear Editor in Chief**

**PLOS ONE**

**After greeting**

I'd like to extend my thanks and appreciation for your kind efforts. This is regarding my manuscript Ref. **Submission ID PONE-D-23-24946R1**, entitled "**Computational analysis of damaging non-synonymous single nucleotide polymorphisms (nsSNPs) on human tyrosinase protein**". I confirm that all corrections, suggestions and queries made by the subject expert and all were given full consideration. All corrections were made in the manuscript file and marked as track change. Our response against each recommendations is mentioned below.

**Reviewer #1:**

The manuscript, entitled "Computational analysis of damaging non-synonymous single nucleotide polymorphisms (nsSNPs) on human tyrosinase protein" is well written and all the sections of the manuscript are presented well and the work done by the authors is new and has scientific significance so this work can be considered for publication after checking these minor points:
**Author Response:** Thanks for appreciation, we have revise the all points raised by the subject expert
**Suggestion 1-** Although it is extremely logical that interactions between the tyrosinase enzyme and compounds known as FDA-approved drugs as inhibitors have been made, I recommend examining the properties of two of the compounds that are claimed to be superior to the drugs determined by the FDA in the literature.

**Author Response:** They have been added in the introduction as suggested. Moreover, a parallel study, we also docked literature-based effective tyrosinase inhibitor compounds and FDA-approved drugs with tyrosinase native and mutant models to see the binding affinity (E_b_) and estimated equilibrium dissociation constant values (K_d_). Therefore, we add the literature based information in introduction section that cover your query.

**Suggestion 2-** Newly synthesized tyrosinase inhibitors should be included in the "INTRODUCTION" section.

**Author Response:** Newly synthesized tyrosinase inhibitors have been added in the introduction as suggested as per recommendation.

**Reviewer #2:**

**Suggestion 1-** The above manuscript is interesting in describing the structural and functional consequences of disease-associated non-synonymous single nucleotide polymorphisms (nsSNPs) in the TYR gene, which encodes tyrosinase. The study highlights the nsSNPs, notably K142M, I151N, M179R, S184L, L189P, and C321R, as particularly deleterious variants, which in general impact the structural integrity and functional behavior of human tyrosinase. The target is of importance; however, as a computational biologist, I am not confident about the outcomes of this study due to a limited and primary-level methodology. Following are some suggestions that need to be addressed before the final decision on the manuscript:

**Author Response:** Thanks for your suggestion to improve the quality of our research. We have incorporate all suggestion and recommendation in revised paper**.**

**Suggestion 2-** The title is very classical; preferably, it should be reworded. Specifically, the main components of the title should be rephrased with some smart, appealing words.

**Author Response:** Title has been reworded as suggested.

**Suggestion 3-** Abstract: The abstract is very plain and incomplete, lacking key content areas, and the research purpose was not properly addressed. The relevance or importance of the research work and the main outcomes were not discussed adequately. See lines 31–35; these lines seem to be an incorrect piece of information. It should be explained in detail regarding the methodological scope. What is the purpose of mentioning lines 37–38? Please explain. Please justify how this study advances the readers’ understanding of the molecular repercussions of the target’s gene and its variants.

**Author Response:** Thanks for suggestion. The abstract has been revised as suggested.

**Suggestion 4-** Keywords: The keywords were not appropriate. Please recheck all selected words.

**Author Response:** The new keywords were added according to the paper scope

**Suggestion 5-** Introduction: This section looks like a mismatch of information. The introduction needs a thorough revision, especially in terms of describing the background of the gene or protein target selected, its clinical role, and its recent epidemiological impact on society. I absolutely missed thorough literature that represents a sound background of information from the present work. I would suggest here that authors should make this section a more comprehensive and updated flow of information that makes readers understand the importance of selected genes and why authors used them for this in silico genomic study. The study rational was missing from this section. Why are authors emphasizing that the main aim of this study is to see the significant role of non-synonymous genetic variations and their impact on the tyrosinase protein`s structure and function.? Specifically, the last paragraph was quite confusing and lacked clarity. The abstract isn’t organized and looks more like a mishmash of information.

**Author Response:** Thanks for suggestion to improve the quality of my paper. We have extensively revised the introduction and follow the reviewer suggestion.

**Suggestion 6-** **Methodology:** The very first sentence starts with “to date." I think it is better to mention the search timeline period. This section was absolutely incomplete. It is quite better if authors segregate the prediction methodologies separately in sub-headings. More importantly, this sub-division will help authors mention gene- or protein-based, sequence- or structure-based assessment methodologies. I found some repetition of details in this section. Although this is an optional suggestion, authors should add a flow chart with a better and more appealing way of presenting it. Please check the following manuscripts for a better reference.
 DOI: 10.1021/acsomega.2c04871
DOI: 10.1021/acsptsci.2c00212
DOI: 10.1021/acs.jcim.0c00488

**Author Response:** Methodology has been revised and improved following the suggestion of reviewer. Moreover, we have followed the above mentioned paper to revise the methodology section.

**Suggestion 7-** **Results:** I don’t accept the results in their present form. The results should be divided and subdivided according to the work scope. These aren’t acceptable in the present form. The graphical representations in the manuscript are of extremely low quality, and the authors are advised to use some smart tools to render high-quality, eye-catching plots of their data.

**Author Response:** Results were also revised as suggested by the reviewer. Furthermore, the results were presented in subsections as suggested. Additionally, we have improve the graphical representation of figures as suggested.

**Suggestion 8-** **Discussion:** It would be better to revise this section. Don’t provide too many details about the tools and methods used in the manuscript; focus more on whether your results are consistent and contribute to your overall findings in relation to previous studies and, finally, your future prospects.

**Author Response:** The discussion part was revised accordingly.

**Suggestion 9-** Citations are missing in some cases.

**Author Response:** The relevant citations were added in revised paper

**Suggestion 10-** Add a proper conclusion section to help readers retain the purpose of the article.

**Author Response:** A separate conclusion section was added in revise paper

**Minor comments:**
1. Cover letter: Please check the name and details of the corresponding author mentioned in the reply to the review letter. It should be the same as listed in the authors’ name list of this paper.

Done as per suggestion
2. Kindly add some missing references.

Done as per suggestion
3. Please refer to the above-recommended manuscripts for guidance on writing, figures, and work methodology.

Done as per suggestion

The authors of this manuscript are grateful to the subject expert and editorial members of the journal, for valuable suggestions to improve the quality of our manuscript. We hope this version is suitable for acceptance and publication.

_________________________

**Shabbir Ahmed**

Corresponding author

[shabbirch983@gmail.com](mailto:shabbirch983@gmail.com)
