# Supplementary material for: Computational analysis of the deleterious non-synonymous single nucleotide polymorphisms (nsSNPs) in TYR gene impacting human tyrosinase protein and the protein stability
Source: PLoS One. 2024 Nov 14;19(11):e0308927. doi: 10.1371/journal.pone.0308927 (PMC11563463; doi:10.1371/journal.pone.0308927)
Supplement: Attachment — Submitted filename: Response latter.docx [file pone.0308927.s005.docx]

**Response latter**

********

July 27^th^ 2024

**Dear Editor in Chief**

**PLOS ONE**

**After greeting**

I'd like to extend my thanks and appreciation for your kind efforts. This is regarding my manuscript Ref. **Submission ID PONE-D-23-24946R2**, entitled " **Computational analysis of the deleterious Non-Synonymous Single Nucleotide Polymorphisms (nsSNPs) in TYR gene impacting human tyrosinase protein and the protein stability**". I confirm that all corrections, suggestions and queries made by the subject expert and all were given full consideration. All corrections were made in the manuscript file and marked as track change. Our response against each recommendations is mentioned below.

**Review Comments to the Author**

Please use the space provided to explain your answers to the questions above. You may also include additional comments for the author, including concerns about dual publication, research ethics, or publication ethics. (Please upload your review as an attachment if it exceeds 20,000 characters)

**Reviewer #1:**

The authors have made the indicated corrections. However, the manuscript can be considered for publication after these minor points:

**Query 1:** Authors should cite some recent reports (2022-2023);new compounds for tyrosinase inhibition studies.
(2023).ChemistrySelect,8(42),e202302936.https://doi.org/10.1002/slct.202302936
(2023). Journal of Biomolecular Structure and Dynamics, 41(15), 7128-7143. https://doi.org/10.1080/07391102.2022.2116600
(2022). Journal of Molecular Structure, 1257, 132641. https://doi.org/10.1016/j.molstruc.2022.132641

(2022). Chemistry & Biodiversity, 19(6), e202200140.

**Response:** Thanks for consideration of our paper. We have added the all suggested references and highlighted in red color

**Reviewer #3:**

There is scientific merit in the manuscript titled “Computational analysis of the deleterious Non-Synonymous Single Nucleotide Polymorphisms (nsSNPs) in TYR gene impacting human Tyrosinase protein and the protein stability”. However, the manuscript should be improved significantly before it can be published.

Major points:
Query 1: The rationale for the molecular docking studies is not established. It is also unclear how the docking results support the predictions of the pathogenicity of the SNPs.

**Response:** Molecular docking allowed us to explore how FDA-approved drugs interact with both wild-type and mutant forms of Tyrosinase. This revealed potential therapeutic compounds that might mitigate the effects of pathogenic mutations. Our study identified specific interactions between proposed drugs and mutant Tyrosinase models, suggesting potential pathways for pharmaceutical intervention. Docking studies serve as a predictive tool to screen and rank potential drug candidates based on their binding affinities. This computational approach is cost-effective and time-efficient compared to experimental high-throughput screening methods. The docking scores and binding affinities provide quantitative measures of how mutations impact the protein's ability to interact with ligands. Mutations that significantly reduce binding affinity or alter binding modes can be indicative of a loss of function or destabilization of the protein, which are hallmarks of pathogenic variants. By visualizing the docking poses, we can identify structural changes in the protein that result from the mutations. These changes can disrupt the active site or other critical regions of the protein, leading to impaired function. For instance, our study showed that mutations like K142M and I151N led to altered binding interactions with FDA-approved drugs, highlighting their potential pathogenic impact.

**Query 2:** None of the studied FDA-approved drugs are present in the crystal structure (PDB ID: 7RK7) used for docking. Thus, there is no control for this docking analysis.

**Addressing the Absence of FDA-Approved Drugs in the Crystal Structure (PDB ID: 7RK7)**

**Response:** The concern regarding the absence of FDA-approved drugs in the crystal structure used for docking is valid. Here are the steps we have taken to address this issue:

**Use of AlphaFold Models:** Given the limitations of available crystal structures, we utilized AlphaFold-predicted models to obtain a more complete and accurate representation of the Tyrosinase protein. This approach allows us to explore the full binding potential of the protein, including regions not covered by the existing crystal structure.

**Comparative Analysis:** We conducted docking studies on both wild-type and mutant models of Tyrosinase to compare the binding interactions. This comparative approach helps highlight the specific effects of the mutations and provides a control for our docking analysis

**Query 3:** This is purely a computational study. Results should further be validated using replicated long molecular dynamics simulations.

**Response:** We appreciate the reviewer's suggestion to validate our computational docking results using long molecular dynamics (MD) simulations. While we acknowledge the value of MD simulations in providing dynamic insights into protein-ligand interactions, it is important to note that this study's primary focus is on the initial identification and characterization of deleterious nsSNPs in the TYR gene and their potential impact on protein stability and drug binding. The findings from our docking studies provide valuable initial insights that can inform more detailed follow-up studies, including MD simulations and experimental validations. Conducting long MD simulations for multiple protein-ligand complexes would require substantial computational resources and time, which are beyond the current scope and budget of this study.

**Query 4:** Substantial language editing will be needed. There are many grammar errors.

**Response:** With the help of expert collogue, we have gone through the manuscript and made significant correction for typos and grammatical errors for the overall improvement of language.

**Reviewer #4:**

 It is a well-planned and presented study.
All the queries raised by reviewers have been answered satisfactorily and manuscript revised accordingly. The manuscript can now be considered for publication.

**Response:** We appreciate the comments made the reviewer and its recommendation for acceptance of this manuscript.

The authors of this manuscript are grateful to the subject expert and editorial members of the journal, for valuable suggestions to improve the quality of our manuscript. We hope this version is suitable for acceptance and publication.

_________________________

**Shabbir Ahmed**

Corresponding author

[shabbirch983@gmail.com](mailto:shabbirch983@gmail.com)
